# Reversible Cerebral Vasoconstriction Syndrome: A Common Occurrence but Rare Diagnosis

**DOI:** 10.7759/cureus.8546

**Published:** 2020-06-10

**Authors:** Brian Grundt, Taryn Bolling, Mark L Ritch

**Affiliations:** 1 Internal Medicine, University of South Florida Morsani College of Medicine, Largo Medical Center, Largo, USA; 2 Neurology, University of South Florida Morsani College of Medicine, Largo Medical Center, Largo, USA

**Keywords:** headache, thunderclap headache, reversible cerebral vasoconstriction syndrome, rcvs, neurology, neuroimaging

## Abstract

Reversible cerebral vasoconstriction syndrome (RCVS) is an under-diagnosed condition that results from reversible segmental and multifocal vasoconstriction of cerebral arteries. It can present with a variety of symptoms including sudden “thunder clap” headaches, neurologic deficits, photophobia, phonophobia, nausea, vomiting, and can mimic life-threatening conditions such as a ruptured intracranial aneurysm, primary angiitis of the central nervous system, and cervical artery dissection. The pathology of this condition is still not fully understood and the etiologies vary, making treatment difficult. Our objective is to draw attention to an under-diagnosed condition with common presenting symptoms.

We present a 60-year-old male with sudden onset of severe headache, left-sided numbness and weakness, blurred vision, ataxia, nausea, and dyspnea. CT and MRI brain showed no evidence of infarct or hemorrhage. CT angiography (CTA) of the head and neck showed a narrow caliber basilar artery. With the patient’s clinical presentation and imaging findings, RCVS was suspected and the patient was started on a calcium channel blocker and glucocorticoids. A repeat CTA of the head and neck was performed after initiation of therapy and showed dilation of the basilar artery. Treatment with verapamil and prednisone was continued and the patient’s symptoms gradually improved. He was discharged to skilled nursing for continued physical therapy.

RCVS is a little-understood, under-diagnosed condition that needs to be considered in patients presenting with headaches and neurologic deficits. Additionally, more research needs to be done to truly understand the etiology of this condition.

## Introduction

Headaches are one of the leading chief complaints that lead to patients presenting to their primary care physician or to the emergency room. The lifelong prevalence of headache among the general population is approximately 96%, with a female predominance [1]. There are many etiologies for headaches, the most common of which are tension-type headache, migraine headache, and cluster headache. Tension-type headaches affect nearly 40 percent of the population, while migraine and cluster headaches generally affect 10 and 1 percent of the adult population, respectively [2]. While headaches themselves are normally benign, the association with “red flag” symptoms and signs prompts the need for further workup to evaluate for more worrisome causes. Red flag symptoms and signs include sudden onset of the worst headache of a patient’s life, focal neurologic signs, neck stiffness, personality changes, ataxia, headache after trauma, and headache that is worse with exercise [3]. In this case, we present a 60-year-old male with sudden onset of the worst headache of his life with associated focal neurologic deficits.

## Case presentation

This is a 60-year-old male with past medical history of hypertension, aortic dilation, cirrhosis, and first degree AV block who presented with complaints of sudden onset severe holocranial headache, nausea, left arm numbness and weakness, blurred vision, ataxia, and dyspnea. The patient denied any head trauma preceding the onset of his symptoms. His vital signs were stable at time of presentation. Initial laboratory studies performed in the emergency room (ER) including complete blood count (CBC), complete metabolic panel (CMP), hemoglobin A1c, urinalysis, and urine drug screen were unremarkable. A CT brain was done which revealed no acute intracranial abnormalities. Teleneurology evaluated the patient in the ER and calculated his National Institute of Health Stroke Scale (NIHSS) to be three. Tissue plasminogen activator (tPA) was recommended, however, the patient refused. He was admitted for further evaluation and neurology consultation.

**Figure 1:**
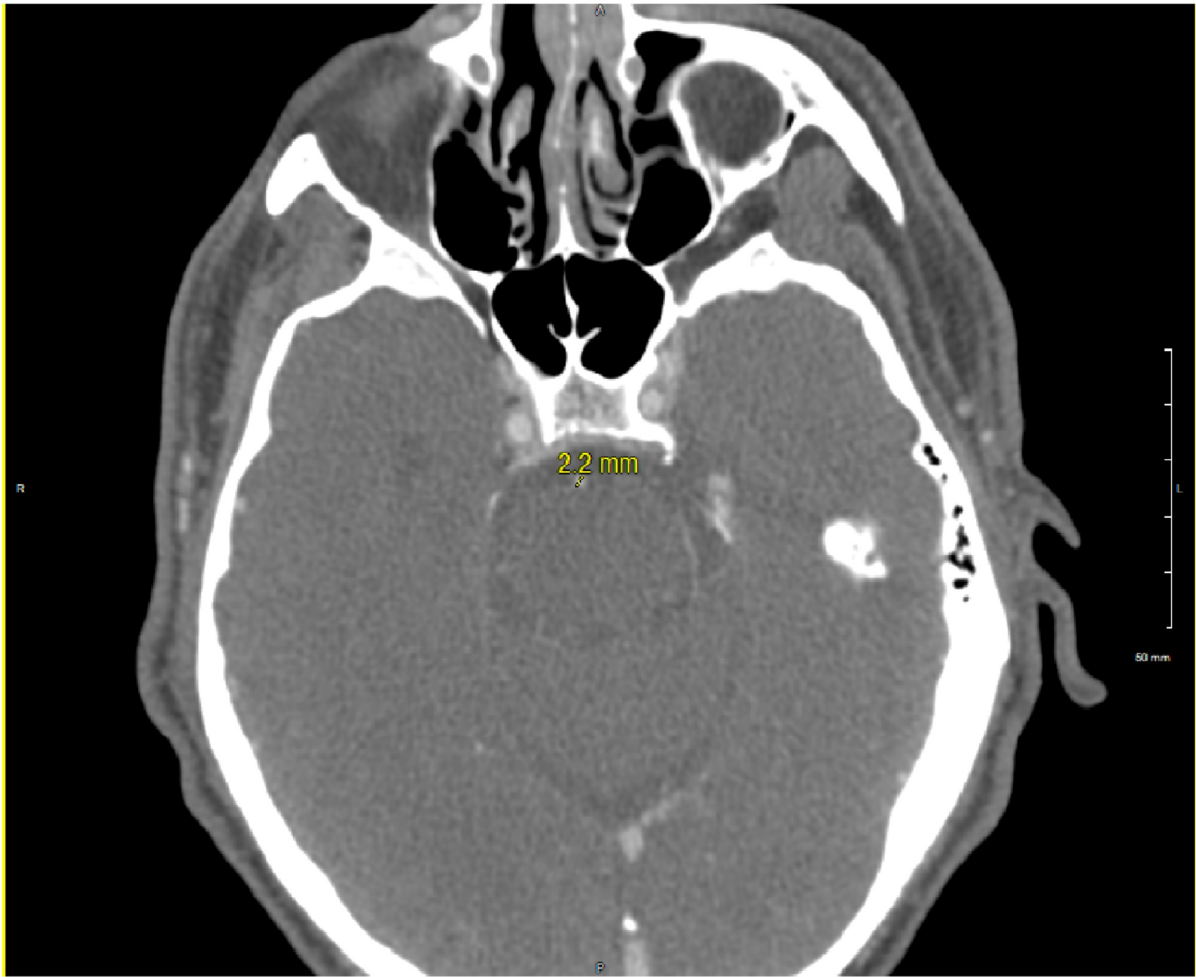
CT angiography (CTA) head performed on hospital day 1 showing a small caliber basilar artery measuring at 2.2 mm.

**Figure 2:**
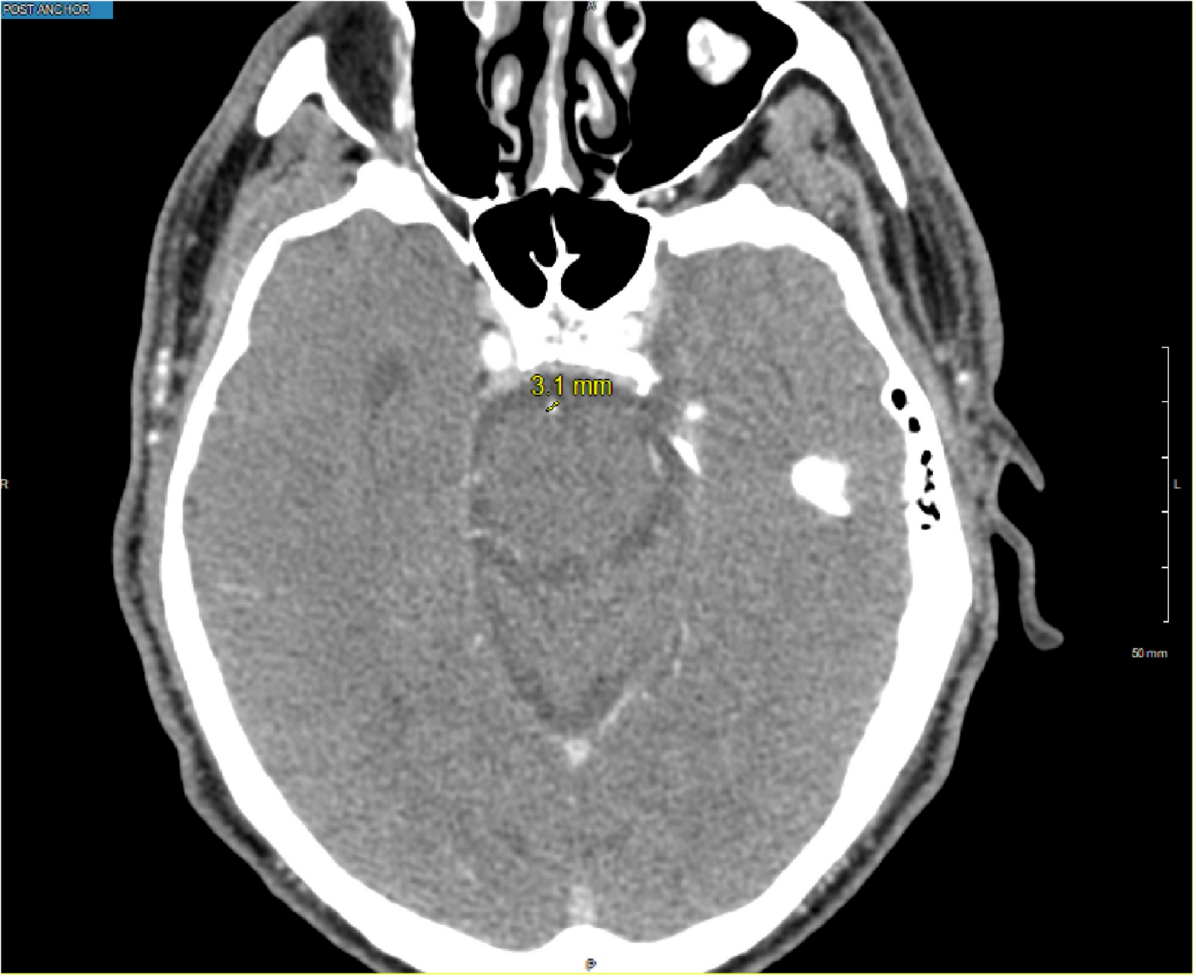
CT angiography (CTA) head performed 48 hours after initiation of calcium channel blocker therapy showing dilation of basilar artery to 3.1 mm.

A CT angiography (CTA) head and neck showed a narrow basilar artery without other apparent vascular abnormalities (Figure 1). MRI brain showed no acute infarct or bleed. Based on the patient’s presenting symptoms and imaging, the diagnosis of reversible cerebral vasoconstriction syndrome was suspected. He was started on a prednisone taper 30 mg twice daily with progressively decreasing doses every three days and nimodipine 60 mg every four hours. However, his blood pressure was unable to tolerate the nimodipine. Therefore, he was switched to verapamil 80 mg three times per day, which he tolerated better. A repeat CTA head was performed 48 hours after initiation of therapy. The repeat CTA showed improvement in the basilar artery narrowing as compared to the initial study (Figure 2).

The patient continued to take verapamil daily. Headaches were noted to persist, although gradual improvement was reported daily. Acetaminophen and hydrocodone were used to supplement headache management. He continued to work with physical therapy and occupational therapy who reported daily improvement in motor and sensory deficits. With his continued progression, the patient was discharged to a skilled nursing facility to continue his rehabilitative therapy with instructions to follow up with neurology for repeat imaging 3-4 weeks after discharge. However, the patient was lost to follow-up.

## Discussion

RCVS is an all-encompassing term to describe a group of disorders characterized by reversible segmental and multifocal vasoconstriction of cerebral arteries. It remains one of the most complex and misunderstood syndromes, partly because it is often confused with other disease processes such as a ruptured intracranial aneurysm, primary angiitis of the central nervous system, and cervical artery dissection [4]. Due to the small number of reported cases, its incidence is unknown. Cases have been reported in patients between ages 10 to 76 years old with the average age of patients affected being approximately 42 years old [5]. RCVS is more common in females compared to males [5]. This differs from the presented case where the patient was a male.

Clinical signs and symptoms which might lead one to suspect RCVS include acute onset of a “thunderclap” headache, that can be classified as a severe bilateral or unilateral head pain, which often begins posteriorly and spreads diffusely [4]. The pain usually peaks in less than one minute and is described as mimicking that of a ruptured aneurysm [5]. Neurologic symptoms may or may not be present. Some of the more common presenting signs and symptoms include nausea, vomiting, photophobia, phonophobia, hemiplegia, ataxia, and aphasia. In this case, the patient presented with symptoms concerning for a stroke with blurred vision, severe sudden headache, and left-sided weakness and paresthesia. His initial images demonstrated discrete narrowing of the basilar artery which differs from many prior cases which show the typical multifocal segmental narrowing of the medium and large arteries in both the anterior and posterior circulation with intermittently dilated segments, also known as the “string of beads” formation [5]. These findings were, however, reversible on repeat imaging, thus leading to the RCVS diagnosis.

It is unknown why the vasoconstriction leading to RCVS occurs. Some researchers feel that the vasoconstriction may be induced by sympathomimetic or serotonergic drugs or catecholamine-secreting tumors, as well as uncontrolled hypertension, oxidative stress, endocrine factors, or trauma [4,6]. This patient reported a history of hypertension. However, he denied any illicit substance or tobacco use, trauma, or endocrine dysfunction. Some reports have found that the risk for RCVS is increased postpartum [5]. Currently, there are no validated criteria for diagnosing RCVS; however, a combination of history, noninvasive imaging, and CSF analysis can help to rule out other etiologies and lead to the diagnosis of RCVS [4].

More research is needed to determine the true pathophysiology behind RCVS and to help uncover possible treatments. Currently, there are no guidelines to direct therapy, but positive outcomes have been reported with calcium channel blockers and a short course of glucocorticoids [4]. Upon diagnosis, this patient was started on a calcium channel blocker and a steroid taper. Many of his symptoms gradually improved over the next few days. He was discharged to inpatient rehabilitation for continued physical therapy. Repeat imaging with CTA or magnetic resonance angiography (MRA) was recommended to monitor continued improvement. Unfortunately, he was lost to follow-up.

Our case affirms that a high index of suspicion is needed to consider a diagnosis of RCVS, which is not a benign condition. Complications of RCVS include ischemic and hemorrhagic stroke, posterior reversible encephalopathy syndrome, and long-term neurologic deficits [7]. The current practice is to treat RCVS using calcium channel blockers and steroids. However, due to the unclear pathophysiology of this condition and lack of significant clinical trials, this regimen is solely based of observational data [4]. Thus, there is a growing need for more study of this condition. It is our hope that this article can inspire future research and prospective trials to determine long-term outcomes, recurrence rates, and further treatments for this mysterious syndrome.

## Conclusions

RCVS is an under-diagnosed condition due to the significant overlap of symptoms with many life-threatening conditions and no definitive diagnostic criteria. Upon ruling out of these fatal conditions, RCVS needs to be considered as an etiology for a patient’s symptoms with appropriate tests being ordered and medications initiated. No current guidelines exist for treatment. However, multiple treatment options are available and further research should be completed to determine additional options.
